# Risk Assessment of Pre-dialysis Chronic Kidney Disease (CKD) Patients for Cardiovascular Disease (CVD) in a Tertiary Hospital in Nigeria: A Case-Controlled Cross-Sectional Study

**DOI:** 10.7759/cureus.36725

**Published:** 2023-03-27

**Authors:** Henry Ovwasa, Henry O Aiwuyo, Ogochukwu A ‪Okoye, Ejiroghene M Umuerri, Austine Obasohan, Evelyn Unuigbe, Nilum Rajora

**Affiliations:** 1 Family Physician, Milk River Health Center, Milk River Alberta, CAN; 2 Internal Medicine, Brookdale University Hospital Medical Center, Brooklyn, USA; 3 Internal Medicine, Delta State University Teaching Hospital (DELSUTH), Oghara, NGA; 4 Medicine, Delta State University, Abraka, NGA; 5 Internal Medicine/Cardiology, Delta State University Teaching Hospital, Oghara, NGA; 6 Medicine, College of Medical Sciences, University of Benin, Benin City, NGA; 7 Internal Medicine, University of Benin Teaching Hospital, Benin City, NGA; 8 Internal Medicine, University of Southwestern Medical Center, Dallas, USA

**Keywords:** tertiary hospital, cvd, ckd patients, pre-dialysis, risk assessment

## Abstract

Introduction: Cardiovascular disease (CVD) is the leading cause of morbidity and mortality in the chronic kidney disease (CKD) population. CKD patients are more likely to die from CVD before ever reaching end-stage renal disease (ESRD). The study, therefore, seeks to identify the prevalence of risk factors of CVD in CKD patients such as systemic hypertension, anemia, dyslipidemia, hypoalbuminemia, albuminuria, and abnormal calcium/phosphate products.

Methods: The study was a case-control cross-sectional study where one hundred fifty hypertensive CKD patients and age- and sex-matched hypertensive non-CKD subjects were consecutively enrolled at the renal unit of Delta State University Teaching Hospital (DELSUTH), Oghara.

Results: The findings of the study revealed the mean ages of cases and controls to be 48.91±11.93 years and 51.0±15.45 years respectively (p-value 0.182). There was an equal number of males and females among the study group and controls (92 males and 58 females) making a male-to-female ratio of 3:2. The prevalence of CVD risk factors such as diabetes mellitus, hypercholesterolemia, hypertriglyceridemia, elevated low-density lipoprotein, anemia, hypocalcemia, hyperphosphatemia, albuminuria, and hypoalbuminemia was significantly higher among the CKD group compared to controls. Similarly, the prevalence of reduced high-density lipoprotein (HDL) was higher among cases than controls, the difference was however not statistically significant.

Conclusion: The study has shown that systemic hypertension, diabetes, anemia, dyslipidemia, hypoalbuminemia, albuminuria, and abnormal calcium/phosphate products increases the risk for CVD in the general population but is more expressed and significant in CKD patients.

## Introduction

Cardiovascular disease (CVD) is the leading cause of morbidity and mortality in patients at every stage of chronic kidney disease (CKD) [1-3]. The incremental risk of CVD in those with CKD compared to the age- and sex-matched general population ranges from 10 to 200-fold, depending on the stage of CKD [4]. The life span of CKD patients is reduced and CVDs such as stroke, acute myocardial infarction, and congestive heart failure, account for premature death in about 50% of dialysis patients [5].

It has been long known that ESRD patients on maintenance hemodialysis (HD) die from cardiovascular causes at a younger age than the normal population [6]. Several Nigerian studies have shown that cardiovascular risk factors and diseases occur early in the course of CKD [7,8].

The life span of CKD patients is generally reduced and CVD accounts for premature death in about 50% of dialysis patients [5]. Even subtle kidney dysfunction has been shown to increase cardiovascular risk among CKD patients [9]. With falling GFR, the prevalence of hypertension increases progressively and is present in 75% to 85% of dialysis patients [10]. Despite the fact that occlusive coronary artery disease (CAD) is often observed in CKD patients, acute myocardial infarction (AMI) accounts for < 10% of cardiovascular death while ‘sudden cardiac death’ is responsible for 60% to 70% of cardiac death in the dialysis patient population [11].

Traditional and non-traditional risk factors have been identified as contributors to the high rate of CVD in CKD. Traditional risk factors are defined as those risk factors in the Framingham Heart Study that have been used to estimate the risk of developing symptomatic ischemic heart disease [12]. Most of the traditional risk factors, such as older age, diabetes mellitus, systemic hypertension, LVH, and low HDL cholesterol, are highly prevalent in CKD [13]. However, the complexity of the relationship between some traditional cardiovascular risk factors and overall mortality in ESRD is worthy of note. For instance, a “reverse epidemiology” or “U-shape” mortality curve has been observed whereby ESRD patients with low cholesterol and low blood pressure paradoxically have increased mortality. This increase in mortality is believed to reflect underlying malnutrition and/or inflammation (in case of low cholesterol) and advanced cardiomyopathy (in case of low blood pressure). Several cross-sectional studies have shown the inadequacy of the Framingham risk equation to sufficiently explain the extent of the CVD risk in the CKD population, which led to the current interpretation that non-traditional risk factors not included in the Framingham risk equation, play a possible important role in promoting CVD in the CKD patients [14,15].

The kidney-specific risk factors include albuminuria, anemia, ECF volume overload, oxidative stress, electrolyte imbalance, inflammation, hyperparathyroidism, hyperhomocysteinemia, and the recently recognized coronary artery calcification gene [16]. There is also increasing evidence that early intervention to combat cardiovascular risk factors such as anemia, albuminuria, hypertension, and calcium and/or phosphate abnormalities may reverse subtle CVDs, particularly LVH [17,18]. The study, therefore, seeks to assess the risk of CVD in pre-dialysis CKD patients.

## Materials and methods

The study is a hospital-based case-control cross-sectional study. One hundred and fifty hypertensive CKD subjects and 150 hypertensive non-CKD age-and sex-matched controls were recruited. The study was done at the Renal and Cardiac Units of Delta State University Teaching Hospital (DELSUTH), Oghara, Delta State, in South-South Nigeria. Oghara is a town in Ethiope West Local Government Area of Delta State with geographic coordinates at 5035’20’’ N606’1’’ E.181 DELSUTH is a state-owned teaching hospital established by the Delta State Government in 2010.

This study evaluated adult patients with CKD with an estimated GFR between 15 to less than 90mL/min/1.73m^2^ (i.e., stages 2 to 4 CKD patients) attending the renal outpatient clinic of DELSUTH. A structured questionnaire was administered by the researcher to the study participants. Inclusion criteria include Consenting CKD patients above 18 years with eGFR between 15 and less than 90mL/mm/1.73m^2^, while exclusion criteria were Unwillingness to participate in the study. Patients with ESRD (i.e., eGFR <15mL/min), dialysis-dependent patients, and patients with primary cardiovascular/valvular diseases such as cardiomyopathies, congenital heart diseases, and rheumatic valvular diseases. Statistical Package for Social Sciences computing program version 22 (SPSS data edition) was used for data management and analysis.

## Results

**Figure 1:**
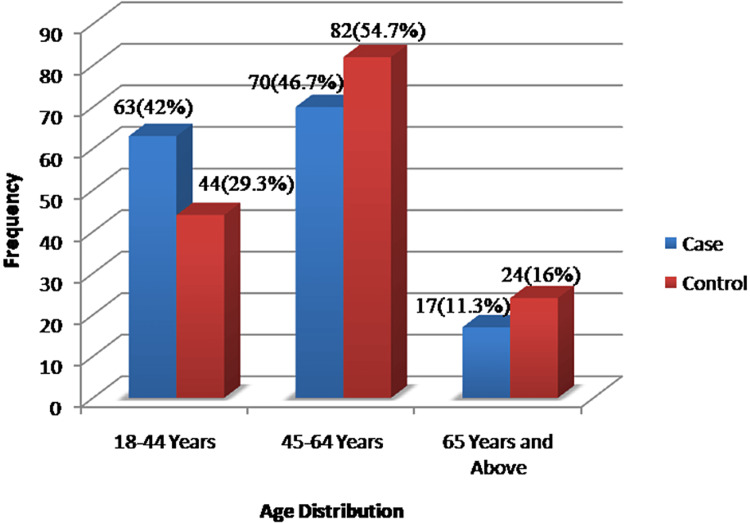
Age distribution among study population x^2^(p-value) = 5.52 (0.063)

One hundred and fifty CKD subjects and 150 hypertensive non-CKD controls who fulfilled the inclusion criteria were recruited. The mean ages of cases and controls were 48.91±11.93 years and 51.0±15.45 years, respectively (p-value 0.182). Eighty-nine percent of the CKD group and 84% of controls were less than 65 years of age. The majority (46.7%) of cases and 54.7% of controls were in the 45-64 years age group. There was no significant difference in the age distribution between cases and controls (x^2^=5.52, p=0.063) (Figure 1). There was an equal number of males and females among cases and controls (92 males and 58 females) making a male-to-female ratio of 3:2 (Figure 1).

Cardiovascular risk profile of cases and controls

Cardiovascular risk factors identified in this study included hypertension, diabetes mellitus, dyslipidemia, anemia, mineral bone disease, albuminuria and hypoalbuminemia. The prevalence of diabetes mellitus, hypercholesterolemia, hypertriglyceridemia, elevated low-density lipoprotein, anemia, hypocalcemia, hyperphosphatemia, albuminuria and hypoalbuminemia were significantly higher among the CKD group compared to controls. Similarly, the prevalence of reduced high-density lipoprotein (HDL) was higher among cases than controls, the difference was, however, not statistically significant (Table 1).

**Table 1:** Prevalence of cardiovascular risk profile of cases and controls *significant at p<0.05 NA-not applicable, TC- total cholesterol, TG-triglyceride, HDL-high density lipoprotein, LDL-low density lipoprotein, Ca/P-calcium phosphate product.

CARDIOVASCULAR RISK FACTOR	CASES, n=150 n (%)	CONTROL, n=150 n (%)	χ^2^	P-value	OR (CI)
Diabetes Mellitus	68(45.3)	23(15.3)	31.94	<0.001*	4.58 (2.56-8.24)
Elevated TC	51(34.0)	16(10.7)	23.54	<0.001*	4.32 (2.24-8.41)
Elevated TG	34(22.7)	3(2.0)	29.63	<0.001*	14.36 (4.09-60-21)
Decreased HDL	55(36.7)	46(30.7)	1.21	0.272	1.31(0.79-2.18)
Elevated LDL	27(18.0)	9(6.0)	10.23	0.001*	3.44 (1.48-8.22)
Anaemia	113(75.3)	17(11.3)	125.10	<0.001*	23.89(12.26-47.16)
Hypocalcaemia	68(45.3)	3(2.0)	77.96	<0.001*	40.63(11.84-167.16)
Hyperphosphatemia	91(60.7)	5(3.3)	113.30	<0.001*	44.73(16.43-131.88)
Elevated Ca/P	8(5.3)	0(0.0)	70.37	<0.001*	undefined
Albuminuria					
Normal	16(13.6)	102(86.4)			
Microalbuminuria	69(46.0)	48(32.0)	131.40	<0.001*	NA
Overt Proteinuria	65(43.3)	0(0.0)			
Hypoalbuminaemia	53(35.3)	0(0.0)	64.37	<0.001*	undefined

## Discussion

The study seeks to assess the risk of CVD in pre-dialysis CKD patients by evaluating systemic hypertension, anemia, dyslipidemia, hypoalbuminemia, albuminuria, and abnormal calcium/phosphate products between the study group and the control. The mean age of cases and controls in this study is 48.91±11.93 years and 51.04±15.45 years, respectively. This finding is comparable with earlier studies that reported the mean age of CKD patients between the third and fifth decades [2,3]. This however contrasts with reports from western countries in which over 50% of the CKD population is aged 65 and above [4,19]. The findings in this study are consistent with the general knowledge that CKD is commoner in young and middle-aged populations in most developing countries. The implication of this observation is that the economic burden of the disease is worse in Sub-Sahara Africa, including Nigeria due to a higher indirect cost in form of man-hours loss at the workplace.

The prevalence of hypertension obtained amongst cases was 78%. This was higher than 70.7% reported in a similar South-Southern Nigerian study [7] but lower than 85.2% reported in a South-Eastern Nigerian study [16] and 96.1% documented by Adejumo et al. in a study done in Benin city [20]. Recent studies have shown a direct association between cardiovascular outcomes and MAP as well as pulse pressure [21,22]. Lowering blood pressure has now been identified as the main target to reduce the incidence of major cardiovascular events in hypertensive CKD patients [23].

Diabetes Mellitus has remained the leading cause of CKD in Western countries there has been an increasing prevalence of DM as an etiology of CKD in developing countries as seen in the index study [24-27]. This rising prevalence of diabetic nephropathy may be a result of epidemiologic transition, westernization, and improvement in the survival of diabetics in SSA, including Nigeria. Diabetes alone has been shown to act as an independent risk factor for several forms of CVD; therefore, the outcome of CVD in diabetic patients is usually worse than in those without diabetes [28-30]. Hence, proper control and treatment of DM, along with aggressive treatment of associated CV risk factors are essential to limiting the rising prevalence and progression of DM and CVD.

The overall prevalence of dyslipidemia in the CKD group (74.7%) was significantly higher than the 12.7% observed in the hypertensive non-CKD controls. The prevalence of elevated Total cholesterol (34%), Triglyceride (22.7%), and LDL-C (18%) were lower than 44%, 26%, and 48% prevalence of elevated TC, TGL, and LDL-C, respectively, reported by Akpan et al. [31] in a South-Western Nigerian study involving mostly CKD stages 4 and 5. However, the overall prevalence reported in the index study was higher than the 60% overall prevalence reported in a South-Southern Nigerian study [20]. The difference in these findings may be a result of variations in diet, lifestyle, and CKD stages. Paradoxically, low rather than high serum cholesterol is associated with poor survival in HD patients [32].

The prevalence of anemia among the CKD group (75.3%) was significantly higher than 11.3% among controls as was expected. This observation is consistent with 77.5% prevalence reported in a study done in Enugu [33] but lower than 88.9% prevalence documented in a South-Western Nigerian study [34]. The difference in these observations may be due to different cut-off points for diagnosing anemia and CKD stages of the study population. However, one observation common to all the studies is the high prevalence of anemia even in early CKD stages, and the frequency, as well as the severity of anemia, increased with deteriorating renal function indices [33,35]. There is a strong association between anemia and LVH, LV dilatation, and risk of sudden cardiac death [36]. Partial correction of anemia with Erythropoietin may result in regression of LVH and renormalization of high cardiac output [37].

Markers of Mineral and Bone Disease analyzed in this study were hypocalcemia, hyperphosphatemia, and elevated CaXPP. The prevalence of hypocalcemia, hyperphosphatemia, and elevated CaXPP in the CKD group was 45.3%, 60.7%, and 5.3%, respectively. This is much lower than the corresponding findings of 59.3%, 75%, and 12.5% by Sanusi et al. in a study done on 40 ESRD patients in Ile-Ife [38] Similarly, Onyemekehia et al. in a cross-sectional study done in UBTH reported even a higher prevalence of hypocalcemia and hyperphosphatemia of 71% and 79%, respectively, but a lower prevalence of elevated Ca X PP of 5% [39]. The disparity observed between these studies may be due to the various definitions, cut-off values used, and differences in the characteristics of the study populations. Hyperphosphatemia and elevated CaXPP have been shown to be strong independent predictors of mortality in the CKD population [40].

Hypoalbuminaemia was present in 35.3% of the CKD group. Surprisingly, none of the controls had hypoalbuminemia. This finding, though comparable with the 35.5% prevalence obtained in a similar study by Adejumo et al. in Benin city [20], is much lower than the 43.2% reported by Agaba et al. [41] in a study done in Jos. The higher prevalence observed in the latter study may be a result of the advanced nature of the disease in that study population. Serum albumin, apart from being a marker of nutrition, is also inversely correlated with inflammation and a predictor of mortality [42].

The prevalence of microalbuminuria in the CKD population in this study (46%) was significantly higher than 32% among controls. Macroalbuminuria was present in 35.3% of cases but none of the controls had macroalbuminuria. This study further re-emphasizes the shortcoming of using dipstick analysis to evaluate urinary albumin excretion. Worsening microalbuminuria confers additional cardiovascular risk because microalbuminuria apart from being a predictor of renal disease and progression of nephropathy in DM patients, it is a surrogate of generalized endothelial dysfunction and increased vascular permeability [43]. Albuminuria has been reported to be independently associated with an increased risk for CV events in both diabetic and non-diabetic populations [13,14].

## Conclusions

This study shows that CVDs occur early in CKD patients and the majority of patients have CVD by the time they present to the Nephrologist. The study has shown that systemic hypertension, diabetes, anemia, dyslipidemia, hypoalbuminemia, albuminuria, and abnormal calcium/phosphate products increases the risk for CVD in the general population, however more expedients in CKD patients.
